# STAT3 activation is required for the antiapoptotic effects of prolactin in cervical cancer cells

**DOI:** 10.1186/s12935-015-0234-9

**Published:** 2015-09-04

**Authors:** Adrián Ramírez de Arellano, Edgar I. Lopez-Pulido, Priscila A. Martínez-Neri, Ciro Estrada Chávez, Renee González Lucano, Mary Fafutis-Morris, A. Aguilar-Lemarroy, José. F. Muñoz-Valle, Ana Laura Pereira-Suárez

**Affiliations:** Ciencias Biomédicas, Centro Universitario de Ciencias de la Salud, Universidad de Guadalajara, Guadalajara, Jalisco Mexico; Laboratorio de Inmunología, Departamento de Fisiología, Centro Universitario de Ciencias de la Salud, Universidad de Guadalajara, Sierra Mojada # 950, Colonia Independencia, 44340 Guadalajara, Jalisco Mexico; Departamento de Clínicas, Centro Universitario de Los Altos, Tepatitlán de Morelos, Jalisco Mexico; Unidad de Biotecnología Médica y Farmacéutica, Centro de Investigación y Asistencia en Tecnología y Diseño del Estado de Jalisco A.C., 44270 Guadalajara, Jalisco Mexico; Instituto Tecnológico y de Estudios Superiores de Monterrey, Campus Guadalajara, Zapopan, Jalisco Mexico; Centro Médico Nacional de Occidente (CMNO), Instituto Mexicano del Seguro Social (IMSS), Guadalajara, Jalisco Mexico; Centro Universitario de Ciencias de la Salud, Instituto de Investigación en Ciencias Biomédicas, Universidad de Guadalajara, Guadalajara, Jalisco Mexico

**Keywords:** PRL, Cervical cancer, STAT3

## Abstract

**Background:**

Prolactin (PRL) has been implicated in the development of different types of cancer. However, signaling pathways might be activated depending on various forms of prolactin receptor (PRLR). JAK/STAT is an important pathway associated with PRL effects. The activation of JAK/STAT pathway might activate antiapoptotic genes that could importantly lead to progression of tumorigenesis. Recently, we have reported that PRL is associated with cell survival by inhibition of apoptosis and the precise activated signaling pathways for this process are still questioned. The purpose of this study was to evaluate the activation of different signaling pathways in response to PRL as well as to identify the induction of antiapoptotic genes.

**Methods:**

Cervical cancer cell lines HeLa, SiHa and C-33 A were stimulated with PRL (200 ng/mL) for 30 and 60 min and non stimulated cells were used to measure basal protein expression. Inhibition assays were performed by using Jak2 specific inhibitor AG490, either alone or in combination with PRL for 48 h. Western blot were carried out to evaluate protein induction of the different signaling pathways and antiapoptotic proteins. Significant effects were determined by using ANOVA test.

**Results:**

STAT3 was significantly activated in cervical cancer lines in comparison with non-tumorigenic keratinocytes HaCaT. No significant differences were found when analyzing MAPK and PI3K signaling pathways. An increase of antiapoptotic genes *Bcl*-*xl*, *Bcl*-*2*, *survivin* and *Mcl*-*1* was observed after stimulus with PRL; however, after inhibition with AG490, the induction of antiapoptotic genes was decreased.

**Conclusion:**

Our data suggests that STAT3 is an important signaling pathway activated by PRL in cervical cancer cells and it modulates the induction of antiapoptotic genes. Blocking STAT3 could represent a possible therapeutic strategy in cervical cancer.

## Background

Prolactin (PRL) is a peptidic hormone with diverse biological effects such as cell proliferation, immunoregulation, osmoregulation and reproduction. PRL is synthesized mainly by the pituitary gland. However, other cell types have the capacity to produce PRL in an autocrine-paracrine manner acting as a growth factor, neurotransmitter and inmunomodulator [1]. These functions are the result of PRL binding to prolactin receptor (PRLR) activating the signaling pathways Janus kinase/Signal transducer and activator of transcription (JAK/STAT), Mitogen-activated protein kinases (MAPK) and phosphoinositide 3 kinase (PI3K). Several forms of PRLR have been described including a long form, an intermediate form and two short forms, each of them with the ability to activate different signaling pathways [2–4].

It has been documented that the activated signaling by PRL/PRLR is associated with tumor development. The association of PRL and its receptor with tumor progression was first established on animal models many decades ago [5]. Recently, there are several studies that involve PRL in the development of different types of cancer such as breast [6–8], prostate [9, 10], ovarian [11] and cervical [12, 13].

With regard to cervical cancer we reported an overexpression of PRLR and the presence of different forms in tissues derived from cervical cancer in comparison with premalignant lesions and healthy tissues [12]. This accumulation in the cytoplasm could be due to alterations in PRLR leading to a decrease of PRLR phosphorylation and subsequent lack of ubiquitination which is associated with the non-degradative process and as a result, the accumulation of PRLR [14]. In addition, we reported that PRL and PRLR induction is associated with cell survival, mainly by inhibition of apoptosis, but not by inducing proliferation, in cervical cancer cell lines [15].

It has been documented that the activation of these signaling pathways induces the induction of target genes associated with proliferation and antiapoptotic effects in breast cancer [16]. However, there are no investigations focused on the induction of antiapoptotic genes in cervical cancer in response to PRL/PRLR.

For this reason, the purpose of this study was to evaluate the signaling pathways involved in the antiapoptotic effect mediated by PRL/PRLR in cervical cancer, as well as the induction of antiapoptotic genes that could be subserving the development of the carcinogenic process.

## Materials and methods

### Reagents

Prolactin (L-4021) was obtained from Sigma-Aldrich^®^. Polyvinylidenedifluoride (PVDF) membranes, enhanced chemi-luminiscence (ECL), Western blotting detection kit (WBKLS0500), and monoclonal antibody anti-actin (Clone C4) were purchased from Merck Millipore^®^ (EMD Millipore Corporation Billerica, MA, USA). Polyclonal antibodies anti-pSTAT3 (Ser 727) sc-13564, anti-STAT3 (H-190) sc-7179, anti-pERK 1/2 (Thr 202) sc-101760, anti-pAKT 1/2/3 (ser 473) sc-101629, anti-AKT 1/2/3 (H-136) sc-8312 as well as the monoclonal antibodies anti-ERK2 (H-9) sc-271451, anti-bcl-xl (H-5) sc-8392 and anti-bcl-2 (C-2) sc-7382 were obtained from Santa Cruz Biotechnology^®^, inc (Santa Cruz, CA). Phospho-p38 (Thr 180/Tyr 182) (D3F9) #4511 and p38 MAPK #9212 were purchased from Cell Signaling Technology^®^ (Danvers, MA, USA). Polyclonal antibody anti-survivin AF886 was purchased from R&D Systems^®^ (R&D Systems, inc. Minneapolis, Mn). Inhibitor for JAK/STAT signaling pathway α-cyano-(3,4-dihydroxy)-*N*-benzylcinnamide (AG490) was dissolved in dimethyl sulfoxide (DMSO) and stored at −20 °C as recommended by the manufacturer. RPMI 1640, DMEM and Fetal Bovine Serum (FBS) were purchased from Gibco^®^, Life technologies (Carlsbad, CA).

### Cell culture

Cervical cancer derived cells (HeLa, SiHa, C-33A), as well as breast cancer derived cells overexpressing PRLR (MCF-7, T-47D), and non-tumorigenic immortalized keratinocytes (HaCaT) from American Type Culture Collection^®^ (University Boulevard Manassas, VA) were used. All cells were grown with RPMI 1640 or DMEM medium supplemented with 5 % of Fetal Bovine Serum (FBS), penicillin G, streptomycin and amphotericin B. Media, FBS, antibiotic and antimicotic were obtained from Gibco^®^, Life technologies (Carlsbad, CA).

Cells were cultured in a jacket-water incubator at 37 °C with an atmosphere of 5 % CO_2_. All cells were grown to reach 80 % confluence so they were used for assays.

The investigation was approved by the ethical investigation and biosecurity committee of the University Center of Health Sciences at the University of Guadalajara (Reference Number C.I. 093/13 CUCS).

### Prolactin stimuli and protein extraction

Cells were seeded in 6-well plates and stimulated with 600 ng of PRL for either 30 or 60 min. Proteins were extracted by using 100 μL RIPA buffer (50 mM Tris, 100 mM NaCl, 1 % NP40, 0.5 % sodiym deoxycolate and 0.1 % sulfate dodecilsulphate (SDS) in addition of a cocktail of protease inhibitors (pepstatin A, aprotinin A, chymostatin, antipain, bestatin and PSMF) as well as a phosphatase inhibitor (Na3VO4) followed by centrifigation at 4 °C for 15 min. Protein extract concentrations were determined by using Lowry method (DC Protein Assay, BioRad Laboratories, Hercules, CA).

### Western blot

Fifty micrograms of total protein extract were mixed with loading buffer and denatured at 95 °C for 5 min. Afterwards they were loaded on 10 % SDS polyacrylamide gels to be resolved. Electrotransference was carried out in polyvinylidene difluoride membranes (BioRad Laboratories, Hercules, CA). Blocking solution was prepared with 5 % of Blotting Grade Blocker (BioRad Laboratories, Hercules, CA) and membranes were incubated in this solution for 2 h. Solutions with primary antibodies were prepared at a dilution of 1:500 and 1:10,000—in the case of β-actin—and membranes were kept overnight followed by either anti-mouse or anti-rabbit secondary antibody solutions (diluted 1:5000). Reveal process was developed with a chemiluminiscence system (Immobilion, Merck Millipore^®^). Microchemi 4.2 was used to reveal membranes and measure optical density as well.

### STAT3 inhibition assay

Cells were seeded in six-well plates for 48 h under three conditions: no stimulus, PRL stimulus, and PRL stimulus plus AG490 inhibitor. During the time of stimuli, cells were kept at 37 °C and 5 % CO_2_. Once the time of stimuli was over, proteins were extracted as described above.

### Apoptotic assay (TUNEL assay)

To carry out TUNEL assay we used the kit APO-BrdU (Invitrogen). Cells were grown for 24 h in eight-well chamber slides seeded with 5 × 10^4^ cells per well were treated with etoposide alone or in combination with PRL or PRL plus AG490 for 24 h and incubated at 37 °C. The slides were washed with PBS and fixed with 4 % paraformaldehyide for 30 min at room temperature. Fixed cells were washed and permeabilized by using 0.2 % Tween 20 for 10 min and then incubated with terminal deoxynucleotidyl transferase and Brd-U for 1 h at 37 °C. After rinsing with PBS, cells were treated with Alexa Fluor 488 dye-labeled anti BrdU antibody at 37 °C for 30 min and mounted with a glass coverslip. Staining of DNA fragmentation was observed with ultraviolet fluorescent microscope (Carl Zeiss) counting at least 200 cells per well.

### Statistical analysis

Data was analyzed by using Graph pad Prism software (Graph pad version 6.01). Significant effects were determined using ANOVA. Statistically significant differences were considered for *p* values <0.05.

## Results

To determine the effect of PRL on the activation of different signaling pathways in the uterine cervical cancer cell line compared to non-tumorigenic immortalized keratinocytes HaCaT, all cell lines were stimulated with PRL during 30 and 60 min. The MCF-7 and T-47D breast cancer cell lines overexpressing PRLR were used. Immunoblotting analysis of cellular proteins was performed to assess the induction of pS727-STAT3, STAT3, pT202-ERK, ERK, pT180/pY182-p38, p38, pS473-Akt and Akt.

### Prolactin induces STAT3 phosphorylation in cervical cell lines

**Fig. 1:**
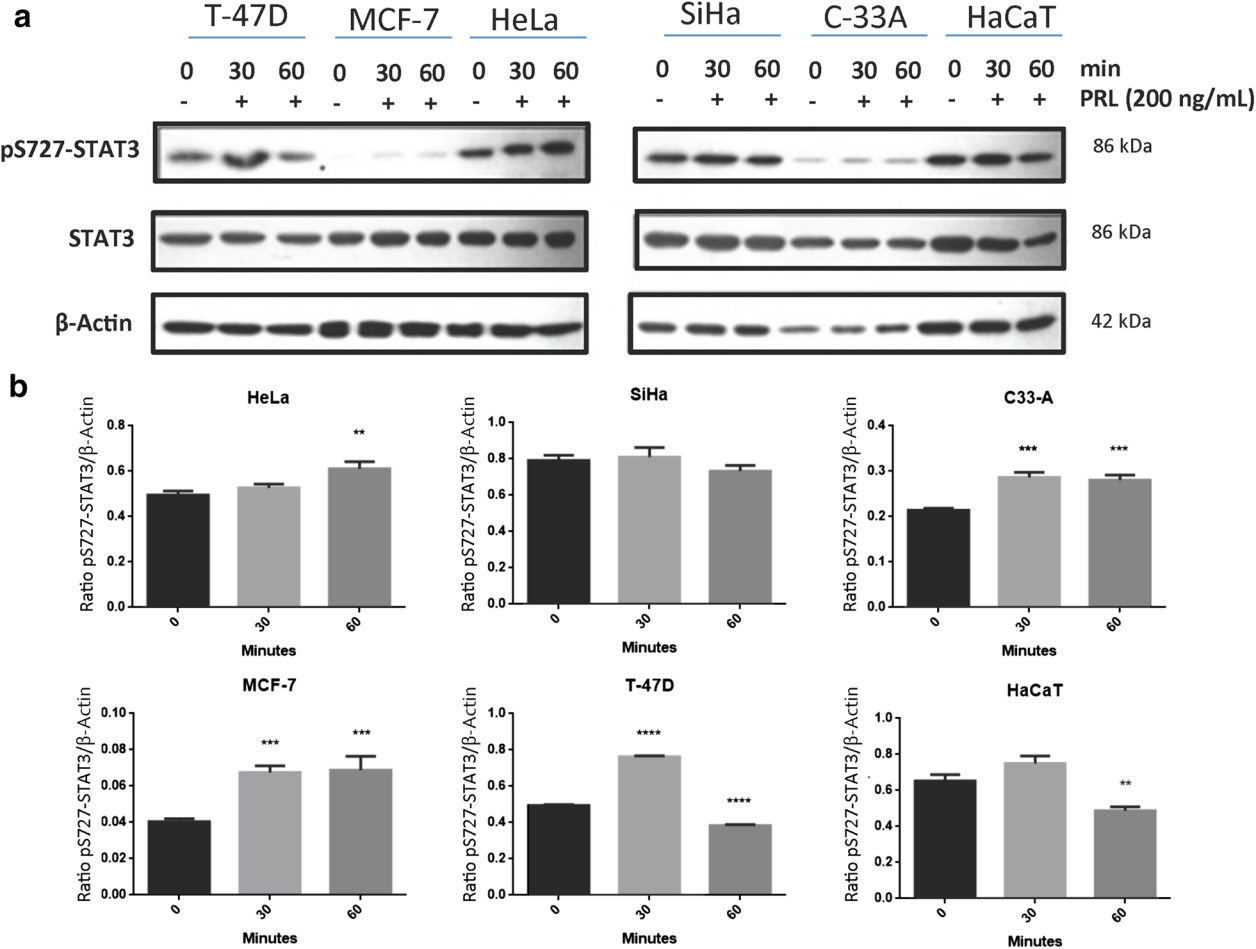
Prolactin induces STAT3 phosphorylation in cervical cancer cell lines by western blot. **a** HeLa, SiHa and C-33 A. Overexpressing PRLR breast cancer cell lines: MCF-7 and T-47D. Non-tumorigenic immortalized keratinocytes: HaCaT. All the cells were treated under three conditions: no stimulus, 30-min stimulus and 60-min stimulus with PRL (200 ng/mL). **b** Induction of pS727-STAT3 by western blot, comparisons were made versus non-stimulated cells, *p < 0.05, **p < 0.01, ***p < 0.001, ****p < 0.0001

The results showed a differential expression pattern of constitutively active pS727-STAT3 among the analyzed cell lines. In comparison to the HPV-negative C-33 A cells, SiHa and HeLa cells demonstrated a higher pS727-STAT3 basal expression. However, treatment with PRL increased pS727-STAT3 induction in HeLa and C-33 A. In MCF-7 and T-47D, increased induction of pS727-STAT3 by the effect of PRL was also observed. In contrast, no differences at 30 min and a decreased pS727-STAT3 expression at 60 min in the HaCaT cell line were observed (Fig. 1).

### Prolactin increases phosphorylation of Akt in the T-47D cell line and decreases in the C-33 A cell line

**Fig. 2:**
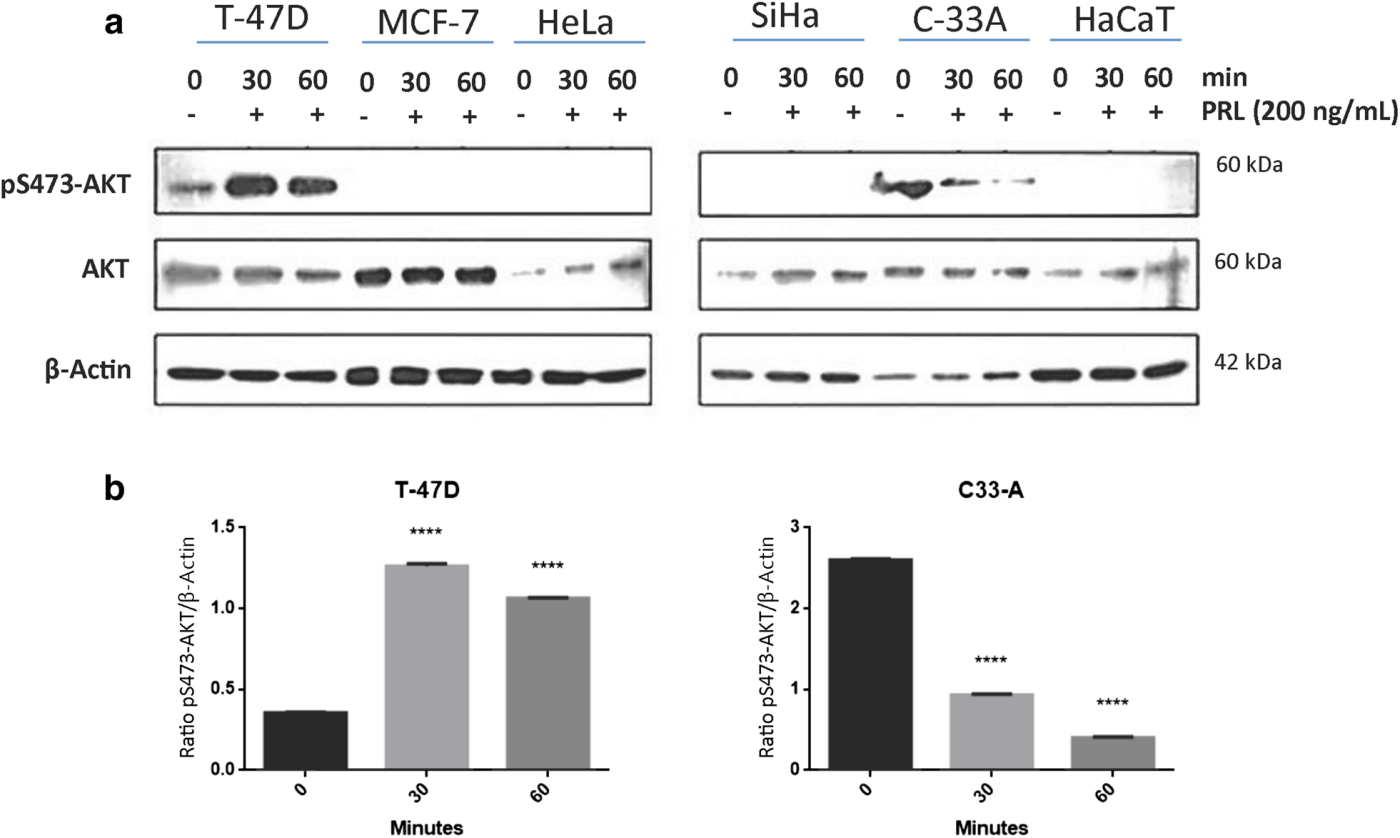
Effect of PRL treatment on AKT phosphorylation in cervical cancer cell lines by western blot. **a** HeLa, SiHa and C-33 A. Overexpressing PRLR breast cancer cell lines: MCF-7 and T-47D. Non-tumorigenic immortalized keratinocytes: HaCaT. All the cells were treated under three conditions: no stimulus, 30-min stimulus and 60-min stimulus with PRL (200 ng/mL). **b** Induction of pS473-Akt by western blot, comparisons were made versus non-stimulated cells, *p < 0.05, **p < 0.01, ***p < 0.001, ****p < 0.0001

The expression of pS473-Akt was only observed in T-47D and C-33 A cell lines. PRL induced activation of Akt in T-47D and the opposite effect was observed in C-33 A (Fig. 2).

### Prolactin modulates the phosphorylation of ERK and p38 in cervical and breast cancer cell lines

**Fig. 3:**
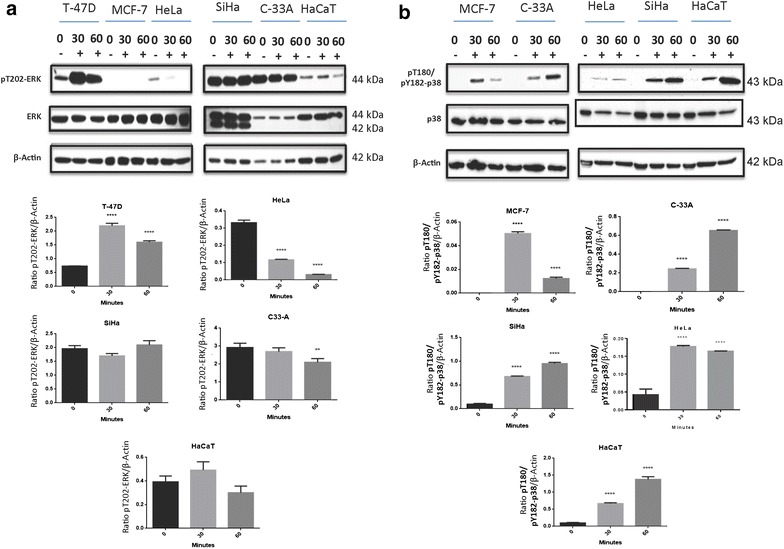
Effect of PRL on MAPK phosphorylation in cervical cancer cell lines by western blot. **a** HeLa, SiHa and C-33 A. Overexpressing PRLR breast cancer cell lines: MCF-7 and T-47D. Non-tumorigenic immortalized keratinocytes: HaCaT. All the cells were treated under three conditions: no stimulus, 30-min stimulus and 60-min stimulus with PRL (200 ng/mL). **b** Induction of pT202-ERK and pT180/pY182-p38 by western blot, comparisons were made versus non-stimulated cells, *p < 0.05, **p < 0.01, ***p < 0.001, ****p < 0.0001

The pT202-ERK expression was observed in cell lines T-47D, HeLa, SiHa, C-33 A and HaCaT. PRL stimulus induced a significant increase in the phosphorylation of ERK in T-47D, both at 30 and 60 min; on the other hand, a downregulation of pT202-ERK induction by PRL was observed in HeLa in a time-dependent manner and in C33 A at 60 min (Fig. 3a). Moreover, a significant increase of phospho-p38 induction modulated by PRL was perceived in cell lines MCF-7, C-33 A, HeLa, SiHa and HaCaT. The peak of p38 activation in MCF-7 and HeLa was observed at 30 min, while in cell lines C-33 A, SiHa and HaCaT, it was at 60 min (Fig. 3b).

### The activation of STAT3 is directly correlated to the induction of anti-apoptotic genes

**Fig. 4:**
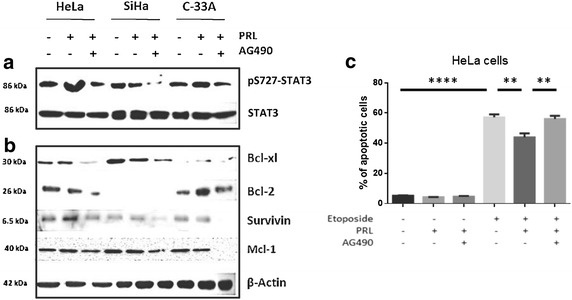
Effect of PRL and AG490 on *bcl*-*xl*, *bcl*-*2*, *survivin* and *Mcl*-*1* induction in cervical cancer cells. HeLa, SiHa and C-33 A were treated with either PRL alone or in combination with AG490 inhibitor. **a** Induction of pS727-STAT3. **b** Induction of antiapoptotic genes *bcl*-*xl*, *bcl*-*2*, *survivin* and *Mcl*-*1.* **c** Apoptosis in HeLa cells after stimuli with Etoposide, PRL and AG490

STAT3 induction in cervical cancer cell lines was the only pathway that showed activation after PRL stimulus compared to HaCaT. Therefore, to test the functional relevance of active STAT3 in the induction of antiapoptotic genes, we inhibited the JAK/STAT pathway by using AG490 inhibitor. In order to evaluate effectively that the signaling pathway had been inhibited, the induction of pS727-STAT3 was assessed. The results showed that the increment in the PRL-mediated phosphorylation of STAT3 in the cell lines HeLa, SiHa and C-33 A was detectable 48 h after treatment and that co-stimulation with the AG490 reduced this effect in the three cervical cancer cell lines (Fig. 4a).

After stimulation with PRL, an increment in the level of Bcl-xl was observed in HeLa, SiHa and C-33 A and the inhibition of STAT3 diminished the induction of this anti-apoptotic molecule in all three analyzed cell lines (Fig. 4b).

On the other hand, the induction of Bcl-2 was increased in C-33 A after treatment with PRL, and inhibition of STAT3 reduced the induction in both HeLa and C-33 A cell lines. In the SiHa cell line, the induction of Bcl-2 was not observed. The induction of survivin was increased in the cell lines HeLa and C-33 A after stimulus with PRL and the inhibition of STAT3 reduced its induction in all three analyzed cell lines (Fig. 4b).

When analyzing Mcl-1 expression we can observe that there is a decrease in its induction when blocking STAT3 signalling in this 3 cell lines (Fig. 4b).

### Apoptosis is directly related to decrease of antiapoptotic genes in HeLa cells

After stimuli with etoposide apoptosis is augmented in comparison to those non stimulated but this increase is diminished when PRL is present in the medium. Yet, after blocking JAK/STAT pathway apoptosis is restablished (Fig. 4c).

## Discussion

The abnormal induction of PRL/PRLR has been associated with the development and progression of various types of cancers such as breast [17], prostate [18, 19], colorectal [20, 21], larynx [22], and hepatocellular [23, 24]. However, few studies have reported the induction of PRL/PRLR in cervical cancer, so their role has been poorly studied. A previous study reported that the presence of PRL is increased in cervical cancer tissue from patients compared with controls [25]. Additionally, an increase in serum PRL levels was reported in a considerable number of patients with cervical cancer [26]. Recently, our research group observed a high induction of PRLR in samples of patients with cervical cancer compared with samples from patients with intraepithelial lesions [12].

Cancer development related to PRL, is mainly described in breast cancer [27, 28]. Some authors have reported that PRL promotes the proliferation of T-47D and MCF-7 cell lines [29]. In contrast, other authors show that PRL does not have any effect on cell proliferation in the breast cancer cell lines MDA-MB-231, T-47D, MCF-7 and Hs578T [8]. These differences may be caused by the use of different techniques and culture conditions as well as different clones for each cell line. Another study, reported that treatment with PRL does not promote proliferation in PC3 and LNCaP cell lines derived from prostate cancer, but has shown a pro-apoptotic effect on the LNCaP cell line which responds to stimulation with androgens [30]. On the other hand, it has been reported that PRL stimuli prevents apoptosis after treatment with C2-ceramide in the breast cancer cell lines T-47D, MCF-7 and Hs578T [8].

Similar results, reported by our research group show that treatment with PRL has a protective effect against etoposide-induced apoptosis by decreasing the number of apoptotic cells in HeLa, SiHa y C33A, compared with the immortal human keratinocyte HaCaT cell line. This suggests that PRL plays an important role in the survival of cervical cell lines [15].

Regarding the activation of signaling pathways that modulate such effects, it has been reported that Jak2 is essential for the proliferative effects of PRL on the induction of breast tumorigenesis, however, deletion of Jak2 after neoplastic transformation, does not have an impact on survival and proliferation of breast cancer cells in culture or in vivo [6].

In the same context, PRL/PRLR signaling has the ability to promote progression and invasion in breast cancer through independent pathways to JAK2/STAT5, such as c-Src, FAK and MAPK. In addition, PRL may promote cell motility and confer resistance to chemotherapy, which contributes to metastasis [28, 31].

In this study, at first instance, we analyzed which signaling pathway is activated on cervical cancer cell lines after the stimulation with PRL; we observed an important increase in STAT3 phosphorylation, compared to the HaCaT cell line; however, no changes were observed in the activation of other signaling pathways.

Previously, and consistent with our results, it has been reported that the signaling pathway that involves STAT3 is activated constitutively in cervical cancer, this was observed in vivo on the tissue from patients as well as in vitro on different cell lines derived from cervical cancer [32].

On the other hand, a strong positive correlation of constitutively active STAT3 with induction of HPV16 E6 and E7 oncoproteins and a negative association with levels of p53 and pRB on HPV16-positive cervical cancer cell lines (SiHa and CaSki) and primary tumor tissues, have been reported [33].

Furthermore, another previous work reported an over-activation of STAT3 observed in tissues from patients with cervical cancer, which is associated with increased induction of anti-apoptotic genes such as *Bcl*-*xl*, *survivin* and *Mcl*-*I* [13].

Our results further demonstrate that PRL increases the induction of *Bcl*-*xl*, *Bcl*-*2*, *survivin* and *Mcl*-*1* antiapoptotic genes. Due to activation of STAT3 and the over-induction of antiapoptotic genes found on cervical cancer cell lines after PRL stimulation, we decided to inhibit the STAT3 activation using the inhibitor AG490; which resulted in an impaired induction of *Bcl*-*xl*, *Bcl*-*2*, *survivin* and *Mcl*-*1*. In order to confirm the functional effect of STAT3 activation on apoptosis of cervical cancer cell lines, TUNEL assays were carried out and we observed that there is a direct correlation between the induction of antiapoptotic genes and apoptosis.

Similar to this finding, the suppression of STAT3 induction or activation on SiHa and Caski was associated with the gradual loss of HPV16 E6 and E7 induction and was accompanied by the loss of cell viability [33].

## Conclusion

Our findings suggest that PRL could be modulating the induction of antiapoptotic genes through STAT3 activation in cervical cancer cells, without discarding the involvement of other alternate routes to those discussed in this paper.
